# COVID-19 Pandemic: What Can the West Learn From the East?

**DOI:** 10.34172/ijhpm.2020.85

**Published:** 2020-05-31

**Authors:** Mostafa Shokoohi, Mehdi Osooli, Saverio Stranges

**Affiliations:** ^1^Dalla Lana School of Public Health, University of Toronto, Toronto, ON, Canada.; ^2^HIV/STI Surveillance Research Center, and WHO Collaborating Center for HIV Surveillance, Institute for Futures Studies in Health, Kerman University of Medical Sciences, Kerman, Iran.; ^3^Center for Primary Health Care Research, Lund University, Malmö, Sweden.; ^4^Department of Epidemiology and Biostatistics, Schulich School of Medicine & Dentistry, Western University, London, ON, Canada.; ^5^Department of Family Medicine, Western University, London, ON, Canada.; ^6^Department of Population Health, Luxembourg Institute of Health, Strassen, Luxembourg.

**Keywords:** COVID-19, Emerging Infectious Diseases, Pandemic Preparedness, Public Health Responses

## Abstract

Differences in public health approaches to control the coronavirus disease 2019 (COVID-19) pandemic could largely explain substantial variations in epidemiological indicators (such as incidence and mortality) between the West and the East. COVID-19 revealed vulnerabilities of most western countries’ healthcare systems in their response to the ongoing public health crisis. Hence, western countries can possibly learn from practices from several East Asian countries regarding infrastructures, epidemiological surveillance and control strategies to mitigate the public health impact of the pandemic. In this paper, we discuss that the lack of rapid and timely *community-centered* approaches, and most importantly weak public health infrastructures, might have resulted in a high number of infected cases and fatalities in many western countries.

A novel coronavirus disease (COVID-19), caused by severe acute respiratory syndrome coronavirus-2 (SARS-CoV-2), emerged in China’s Wuhan in late 2019. A few weeks later, the virus spread all around the world. On March 11, 2020, the World Health Organization (WHO) characterized COVID-19 as a pandemic. As of May 19, 2020, a total number of 473 458 confirmed COVID-19 cases and 316 169 confirmed deaths has been reported globally.^1^

In the meantime, most high-income western countries have been severely hit by the COVID-19 pandemic, resulting in a rapid surge in the number of infected cases. For example, as of May 19, 2020, six western countries including the United States, the United Kingdom, Spain, Italy, Germany, and France accounted for more than half of the global confirmed COVID-19 cases (ie, 2 497 125 out of 4 731 458) as well as for above two-thirds of COVID-19 deaths (ie, 219 981 out of 316 169), respectively.^1^ Adding other western countries such as Belgium, the Netherlands, Canada, Switzerland, Portugal, Austria, and Sweden to this list further demonstrates the devastating impact of this pandemic on western countries. Such a circumstance appears to conflict with the considerable progress achieved in western countries in control of communicable diseases over the past several decades. While the health systems of most western countries have prioritized hospital-centered management of non-communicable diseases, their capacity to prevent and control emerging infectious diseases has not been given adequate attention, with relatively limited public investments to strengthen epidemiological surveillance systems in the community.^2^ In case of the ongoing COVID-19 pandemic, most western countries have missed the boat by not using the golden window period at the early days of the spread of epidemic that the East Asian countries used to halt the COVID-19 epidemic. Here, we discuss that a lack of pandemic preparedness in western countries necessitates them to strengthen early responses and *community- centered* public health systems, which are in place in some of the East Asian countries.

## COVID-19 Responses in the East

East Asian countries have been suggested as the most successful examples for curbing the COVID-19 epidemic and reducing its community transmission. Among other factors, a major contributor of such achievements is possibly due to their rapid recognition of the COVID-19 threat, and quick responses to the epidemic. Here, we present the responses to COVID-19 outbreak in some of these countries including South Korea, Vietnam, Taiwan, and Hong Kong, and highlight that their timely community-centered, public health strategies are the key for their success.

*South Korea*’s response to the epidemic has been considered as one of the most successful models worldwide. Along with the most common public health interventions such as physical distancing and school closure, South Korea implemented multiple important community-centered strategies, including but not limited to, rapid expansion of the diagnostic capacities in the communities, widespread testing and screening programs, along with ongoing and extensive efforts in isolating infected cases and tracing and putting their contacts in quarantine. Being well-prepared, as well as the rapid implementation of the public health responses in the early stages have effectively contributed to the control of the epidemic in South Korea. In addition, using advanced information technologies helped the authorities trace the contacts extensively. Innovative screening tests (eg, drive-through and walk-in screening and testing sites) were established in the early stages of the epidemic.^3,4^

*Vietnam* achieved positive outcomes in controlling the epidemic mainly due to the early recognition of the COVID-19 threat and a swift response. Even though wide-ranging testing programs in the community was not affordable, Vietnam implemented other whole-of-society approaches including case identification, case isolation, extensive contact tracing, quarantine of suspected cases, and mass masking aiming at reducing community transmissions and controlling the infections at sources; all of which were implemented from the early days of the spread of the epidemic. Advanced information technology was also incorporated into the responses in Vietnam; for example, people were made to report the health status of themselves and their family members to the national response authorities through a mobile application.^5,6^

In*Taiwan,* multiple well-trained and experienced teams swiftly recognized the COVID-19 crisis after the identification of the first COVID-19 cases, and responded accordingly. Taiwan enhanced proactive case finding using advanced technology, robust and comprehensive contact tracing, encouraged quarantine of suspicious cases, reassured and educated the public while preventing the circulation of misinformation, allocated resources (eg, mass mask production) and addressed COVID-19-related stigma for the affected cases. Taiwan became an example for “how a society can quickly respond to a crisis.”^7,8^

In*Hong Kong*, reinforced preparedness to public health emergencies enabled a nationwide response to the COVID-19 epidemic even before the diagnosis of the first case in the country. Multiple containment approaches including aggressive contact tracing, testing, quarantine of the suspected cases, screening, and surveillance programs were simultaneously implemented.^9,10^

## COVID-19 Responses in the West

As mentioned above, most western countries have been hardly hit by the COVID-19 pandemic. While many western countries have also implemented public health measures such as border control, social/physical distancing programs, testing and contact tracing, majority of them have not been as timely and successful as the East countries.^11^ The less successful management of the COVID-19 in the West could be largely explained by their delayed response and focusing on *patient- centered* strategies and case management in the hospital settings. Of course, among western countries, there are some exceptions; for example, Germany is of those well-praised western countries where the COVID-19 pandemic have been effectively managed. Below, we first explain the reasons for the success of Germany, and then shortly discuss the story of other countries such as the United States, the United Kingdom, Italy, Spain and France in combating the COVID-19 epidemic.

In the European region, *Germany* is an exemplary, much-praised country with their responses to the COVID-19 epidemic. Given its large number of affected cases (ie, 175 210 by May 19, 2020), only 8007 deaths were reported (ie, case-fatality rate [CFR] = 4.5%).^1^ The CFR in Germany is much lower than other countries in Europe, such as Belgium, the United Kingdom, Italy, Spain, and France, as well as the United States.^1,11^ Although countries’ demographic and socioeconomic profile should be also taken into account for the COVID-19 fatality rates,^11^ Germany’s low CFR could be better explained by their early responses through widespread testing programs among a wide-ranging number of people in the community. Compared to other countries conducting targeted testing for either their vulnerable populations (eg, elderly people) or individuals with severe health status, Germany’s extensive testing policy even identified milder cases in non-vulnerable groups (eg, younger ages). Additionally, unlike many other countries, Germany expanded testing laboratories across the country and did not purely rely on the central laboratories. Furthermore, it has been suggested that unlike other countries, Germany did not report considerable community transmission among senior populations, who have been found to be one of the most vulnerable groups. However, Germany like the East countries started the COVID-19 responses much quicker than other western countries to slow down the circulation of the epidemic, as a result of a strong political leadership and commitment.^12^

Some of the other western countries, including *Italy*, *France*, *Spain*, *Belgium*, *Sweden, the United Kingdom*, and *the United States* has not been considered as successful combating the COVID-19 pandemic. These countries have commonly given a substantially late response to the epidemic. For example, Italy implemented more intensified public health response interventions including restricted movement of people and social activities in its most affected region, Lombardy, on March 8, when approximately 12 000 confirmed cases and 800 deaths were reported.^13^ France, Spain, and Italy implemented total country lockdown in early-mid March but weeks after their first cases of COVID-19. Neither of the three countries initiated mass testing, while France had to revisit and extend their testing policy as a potential solution to end lockdown.^14^ In the United States and the United Kingdom, the political leaders hampered the timely response to COVID-19 pandemic due underestimating negative impacts of the viral spread in the community and ignoring the advice of their scientific advisors. Both countries came to implement total lockdown and substantial number of tests, but very late. The final example, Sweden, remains an outlier in handling COVID-19 among Western countries due to a relaxed mitigation strategy. The government of Sweden recommended the citizens to keep social distance, work from home, and avoid establishing or participating large gatherings, and performed limited number of tests.

## The Right Strategies in the Right Time

Supported by the evidence,^9,15^ the deployment of community-centered strategies to detect asymptomatic cases and reduce community transmission is considered the Achilles’ heel of control of fast-spreading infectious pandemics, while symptom-based screening and management of infected cases alone are prone to fail to control community transmission. The lack of such timely, coordinated community- and public health-centered approaches in responding to the global health crisis has the potential to overburden hospitals and healthcare systems,^11^ and lead to a cumulative number of patients and deaths. Besides, without an effective community approach, the pandemics, together with ensuing economic and societal challenges, could aggravate disparities either socioeconomically or via accessing appropriate healthcare services, in particular, among the most vulnerable subpopulations.

The emerging epidemics and pandemics have signified their capacity to become national and international threats, with the COVID-19 pandemic as one such threat providing us with several excruciating lessons. To reduce their burden, western healthcare systems should advance their health emergency preparedness and timely responses, reinforcing intersectoral collaborations in managing resources, and strengthening community responses with stronger public health infrastructures and epidemiological surveillance systems. Community-based approaches—implemented at the right time—are vital to reduce community transmissions and manage the response for pandemics until an effective vaccine becomes available. Trained staff are an essential part of outbreak investigation and control programs. There is a need to boost training in infectious disease epidemiology, as well as in control and surveillance of communicable diseases and emerging infectious diseases. We also agree with an existing body of evidence underscoring the need for promoting international partnership in mitigation and suppression strategies to fight epidemics, expanding national and international investments in prevention and control of emerging infectious diseases, allocating an adequate supply of scarce resources and facilities (eg, personal protective equipment), increasing staffing capacities, enhancing collaborative research, and transparently sharing data during the pandemics.^2^ In addition, as supported by evidence from South Korea^4^ and Taiwan,^7^ utilizing the advanced information technology system for either tracing cases or aggregating critical data are essential in the containment process of the epidemics.

## Conclusion

Emerging infectious diseases such as COVID-19 require timely and rapid community-centered public health responses, epidemiological surveillance, and control strategies to efficiently contain their spread and reduce their associated burden. Health systems in the Western countries need to design and pilot such public health-oriented response programs to be implemented swiftly and at large scale when needed. Health policy-makers and leaders must build and maintain strong ties with the rest of the political system in their nations to gain support and be trusted when early and sufficient measures are needed to save lives.

## Ethical issues

Not applicable.

## Competing interests

Authors declare that they have no competing interests.

## Authors’ contributions

MS and SS conceptualized the manuscript. MS prepared the first draft. MO significantly helped in writing and revising the manuscript. MO and SS improved the manuscript for content and style.

## Authors’ affiliations

^1^Dalla Lana School of Public Health, University of Toronto, Toronto, ON, Canada. ^2^HIV/STI Surveillance Research Center, and WHO Collaborating Center for HIV Surveillance, Institute for Futures Studies in Health, Kerman University of Medical Sciences, Kerman, Iran. ^3^Center for Primary Health Care Research, Lund University, Malmö, Sweden. ^4^Department of Epidemiology and Biostatistics, Schulich School of Medicine & Dentistry, Western University, London, ON, Canada. ^5^Department of Family Medicine, Western University, London, ON, Canada. ^6^Department of Population Health, Luxembourg Institute of Health, Strassen, Luxembourg.
